# ASSOCIATION OF SENSORY AND COGNITIVE IMPAIRMENT WITH HEALTHCARE UTILIZATION AND COST IN MEDICARE BENEFICIARIES

**DOI:** 10.1093/geroni/igz038.170

**Published:** 2019-11-08

**Authors:** William Deardorff, Phillip liu, Richard Sloane, Courtney Van Houtven, Susan N Hastings, Harvey J Cohen, Heather E Whitson

**Affiliations:** 1 Duke University School of Medicine, Durham, North Carolina, United States; 2 Duke University, Durham, North Carolina, United States; 3 Center for the Study of Aging, Duke University Medical Center, Durham, North Carolina, United States

## Abstract

The combination of sensory and cognitive impairment is increasingly prevalent among older adults and may be an important driver of healthcare cost due to functional disability and reduced self-care. This presentation focuses on the relationship between hearing and/or vision impairment and cognitive impairment with hospital admissions and healthcare cost using data from the Medicare Current Beneficiary Survey, a nationally representative sample of community-dwelling adults. We show that the presence of sensory impairment is associated with increased risk of hospitalization regardless of dementia status. In adjusted models, annual total healthcare costs were generally higher among those with sensory impairments compared to those without sensory impairments. We will also discuss work related to the development of a prognostic model that provides estimates of hospitalization risk among older adults with self-reported hearing and/or vision impairment. This model may help inform allocation of health care resources to those at highest risk for adverse outcomes.

